# Family Caregiving Across the Life Course: Patterns, Consequences and Support Strategies

**DOI:** 10.1093/geroni/igaf122.920

**Published:** 2025-12-31

**Authors:** Yiqing Qian, Katherine Miller, Katherine Ornstein

## Abstract

Caregiving for aging families represents an important life transition, intersecting with many life domains such as family relationships, education, employment, and social engagement. Despite its long-term implications on health and well-being, fewer studies have investigated caregiving as a dynamic lifespan experience and its impacts on late-life outcomes. This symposium integrates multiple data sources and approaches to build evidence on family caregiving across the life course and seek solutions to enhance family caregiver support through policy and technology. First, we will examine the life histories of family caregivers using panel data from the Health and Retirement Study. Dr. Miller describes the educational and economic status of youth caregivers as older adults compared to individuals who never become caregivers. Dr. Johnson explores how early-life relationships with parents and grandparents differ between caregivers and those who never become caregivers. Next, we focus on gendered patterns of caregiving for older adults and their implications for work and health using nationally representative data from the National Study of Caregiving. Dr. Patterson reports national trends regarding the impact of caregiving on work by gender. Dr. Gimm examines gender differences in caregiving tasks and strain among older spousal caregivers. Finally, with a focus on solutions to support caregivers in self-care, social participation, and navigating emerging challenges, Dr. Hu presents the pilot testing of a novel user-centered mobile health application to assist caregivers. Dr. Ornstein, as the discussant, will synthesize the implications for research and policies to support the growing needs of diverse family caregivers in the US.

